# The Role of Prophylaxis and Dietotherapy in Gynecology in the Context of the Interdisciplinary Nature of Genital Discomfort—A Pilot Report

**DOI:** 10.3390/jcm14248863

**Published:** 2025-12-15

**Authors:** Grażyna Jarząbek-Bielecka, Agata Puszcz, Mariola Pawlaczyk, Katarzyna Plagens-Rotman, Małgorzata Mizgier, Magdalena Pisarska-Krawczyk, Jakub Mroczyk, Witold Kędzia

**Affiliations:** 1 Gynecology Clinic, Poznan University of Medical Sciences, 60-535 Poznan, Poland; grajarz@tlen.pl (G.J.-B.); kpr.pielegniarstwopolskie@interia.eu (K.P.-R.);; 2 Sexology and Clinical Psychology Student Research Group, Poznan University of Medical Sciences, 60-812 Poznan, Poland; 3 Katedra i Zakład Kosmetologii Praktycznej i Profilaktyki Chorób Skóry (KPPS), Poznan University of Medical Sciences, 60-781 Poznan, Poland; mariolapawlaczyk@ump.edu.pl; 4 Zakład Dietetyki Sportowej, Katedra Dietetyki, Wydział Nauk o Zdrowiu, Akademia Wychowania Fizycznego w Poznaniu, 61-871 Poznan, Poland; mizgier@awf.poznan.pl; 5 Nursing Department, Uniwersytet Kaliski, 62-800 Kalisz, Poland; magmp@op.pl

**Keywords:** vulvar diseases, pruritus vulvae, genital diseases, female, gynecology

## Abstract

**Background/Objectives:** Genital discomfort, manifested by vulvar itching and burning, is a frequent complaint among women of all ages and has multifactorial origins—including dermatoses, infections, allergies, and hormonal disorders. The study aimed to determine whether selected medical history factors—age, obstetric history, and body mass index (BMI)—influence the frequency of genital discomfort as a reason for gynecological consultation. **Methods**: A pilot study included 288 female patients aged 11–91 years who presented to outpatient gynecological clinics between September 2018 and February 2025 with symptoms of vulvar itching and genital discomfort. Qualitative data were expressed as numbers and percentages, and age was described using mean, median, quartiles, and range. Associations between categorical variables were assessed using Pearson’s chi-square test, with statistical significance set at *p* < 0.05. **Results**: The mean age of patients was 47.4 ± 20.3 years. Most were diagnosed with ICD-10 code N90 (82.6%), while 17.4% had N76. Genital discomfort was most frequently reported by women aged 41–50 years (*p* < 0.0001). Comorbidities (*p* < 0.0001) and obstetric history (*p* < 0.0001) significantly influenced the occurrence of genital discomfort, which was more prevalent among women with chronic conditions and those who had been pregnant. No significant associations were found with BMI (*p* = 0.2353) or menopausal status (*p* = 0.3458). **Conclusions**: Genital discomfort is a common and multifactorial condition requiring an interdisciplinary diagnostic and therapeutic approach. Collaboration among gynecologists, dermatologists, endocrinologists, and dietitians is crucial for effective management and prevention.

## 1. Introduction

In the contemporary management of chronic pain syndromes in gynecology, a holistic approach is recommended, integrating aerobic physical activity, dietary interventions and supplementation, relaxation techniques, and appropriate pharmacotherapy and physiotherapy. Particular emphasis should be placed on sexual health, with treatment strategies directed toward the identification and management of sexual dysfunctions secondary to the pain syndrome [1].

In the present study, the “genital discomfort” reported by the patients most closely corresponded to the diagnostic criteria for vulvar pain disorders (VPD), in particular, noninflammatory disorders of vulva and perineum [2,3] (ICD-10 code: N90) and vulvovaginitis (ICD-10 code: N76). The term “other noninflammatory disorders of the vulva and perineum” in ICD-10 (N90.x) encompasses a heterogeneous group of conditions, including dysplastic lesions, leukoplakia, atrophy, hypertrophy, and vulvar cysts. Vulvovaginitis is defined as an inflammatory condition involving the vulva and vagina, manifested by vaginal discharge and subjective symptoms such as pruritus, burning, or pain, and attributable to infectious or non-infectious factors [4]. In the group of sexually active women, both vulvovaginitis and non-inflammatory disorders are associated with dyspareunia and decreased libido, becoming a partnership problem that often requires sexological couples therapy [5]. Among postmenopausal and elderly patients, due to the risk of vulvar cancer, it is not only a gynecological issue but also an important dermatological and oncological concern [6].

The problem of genital discomfort may have multiple causes—from dermatoses, fungal and bacterial infections, and allergies (vulvovaginitis), to hormonal disorders and obstetric complications (VPDs) [5]. Genital discomfort affects patients across all age groups and has become a significant concern also in developmental-age gynecology (pediatric and adolescent gynecology) [7].

In this pilot analysis, typical correlations between discomfort and variables such as age group, comorbidities, pregnancy status, BMI (underweight/normal/overweight/obese), or menopausal status (<48 years/≥48 years) were not examined. Instead, an attempt was made to determine whether any specific subcategory predominated among the studied gynecological patients. Because both the outcome and the explanatory variables were categorical, Pearson’s chi-square test was applied as a non-parametric procedure for comparing observed and expected frequencies in contingency tables. The test was used to assess whether the distribution of genital discomfort across the predefined subcategories differed from the distribution that would be expected by chance. In this way, the statistical analysis complemented the descriptive objective of identifying potentially predominant groups, without implying causal relationships or “influences” between variables.

Given the widespread nature of the problem of genital discomfort (vulvar itching), the aim of this study was to determine whether, and to what extent, selected factors from the medical history—such as age, obstetric history, and body mass index (BMI)—influence the frequency of this condition as a reason for visiting a gynecologist.

## 2. Materials and Methods

The study included 288 gynecological patients aged 11 to 91 years, who presented to the gynecological outpatient clinics with symptoms of genital discomfort between 26 September 2018, and 18 February 2025. The size of the study group was determined by the number of patients who presented to the outpatient clinic and received a diagnosis of other inflammation of vagina and vulva (ICD-10 code: N76) or other noninflammatory disorders of the vulva and perineum (ICD-10 code: N90). It should be emphasized that this study did not analyze the subgroup of girls attending pediatric gynecology clinics, in whom such discomfort was an additional but not the primary problem; this group was analyzed separately in another study.

Qualitative data were described using numbers and frequencies, while age (in years) was characterized using the arithmetic mean, standard deviation (SD), median, lower quartile (Q25), upper quartile (Q75), minimum (Min.), and maximum (Max.) values.

To verify the equality of patient subgroup sizes, the Pearson chi-square test was applied. The Pearson chi-square goodness-of-fit test applied in the analysis met all required assumptions: the variables were measured on a nominal scale (with any potential ordering of categories disregarded); Cochran’s rule was satisfied, with no expected cell count below 1 and no more than 20% of expected counts below 5; and the total of observed frequencies equaled the total of expected frequencies.

A value of *p* < 0.05 was considered statistically significant. Statistical analyses were performed using the STATISTICA 10 PL software package.

## 3. Results

### 3.1. Characteristics of the Study Group

The patients’ ages ranged from 11 to 91 years. The mean age of the women was 47.4 ± 20.3 years. Half of the study participants were 46 years old or younger. Additionally, 25% of the patients were 30 years old or younger, while 75% were 67 years old or younger (Table 1).

In the vast majority of patients, the diagnosis was N90—other noninflammatory disorders of the vulva and perineum, identified in 238 women (82.6%) (Table 2). The remaining patients were diagnosed with N76—other inflammation of the vagina and vulva, observed in 50 women (17.4%).

### 3.2. Verification of Hypotheses

#### 3.2.1. Is There a Relationship Between the Occurrence of Genital Discomfort and Patients’ Age or Comorbidities?

**Figure 1:**
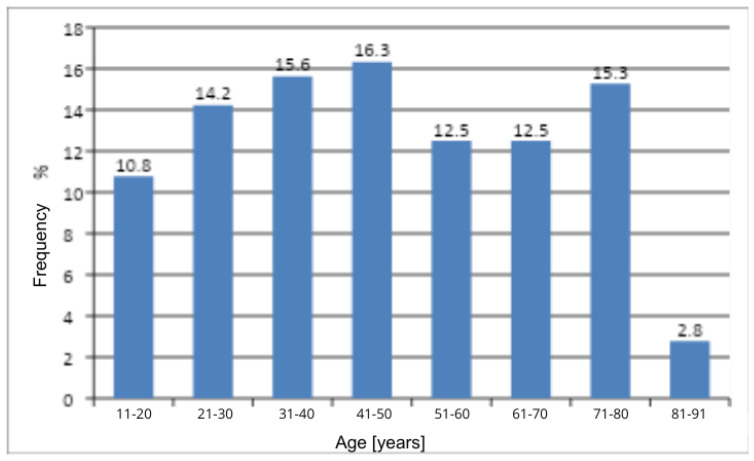
Age Groups of Women Experiencing Genital Discomfort.

The Pearson chi-square test revealed a statistically significant difference in the distribution of women across age categories (*p* < 0.0001) (Table 3). The most numerous group consisted of women aged 41–50 years (Figure 1). Age was found to influence the occurrence of genital discomfort, which was most frequently reported by women aged 41–50 years and least frequently by women aged 81–91 years.

**Figure 2:**
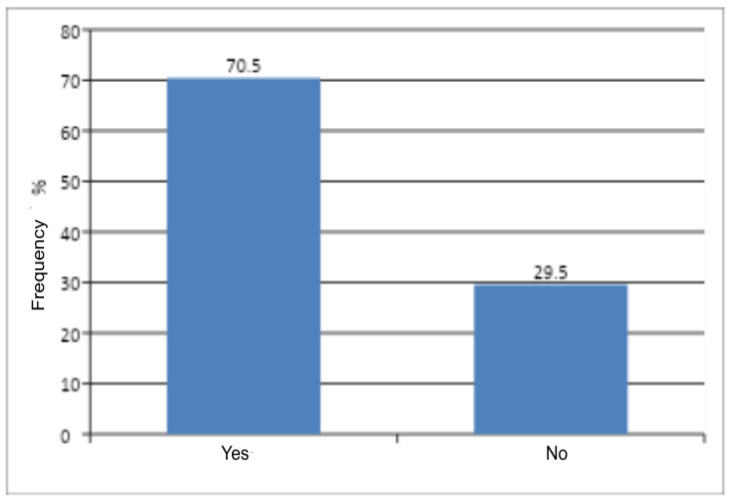
Occurrence of comorbidities among women.

The Pearson chi-square test revealed a statistically significant difference between the number of women with comorbidities and those without such conditions (*p* < 0.0001) (Table 4). Women with comorbidities predominated in the study group (Figure 2). Comorbidities included: metabolic disorders, psychiatric conditions, thyroid diseases, and diseases of the cardiovascular, nervous, respiratory, gastrointestinal, and urinary systems. The presence of comorbidities was found to influence the occurrence of genital discomfort—women with comorbid conditions experienced genital discomfort more frequently.

#### 3.2.2. Is There a Difference in the Frequency of Genital Discomfort Between Women Who Have Ever Been Pregnant and Those Who Have Not?

**Figure 3:**
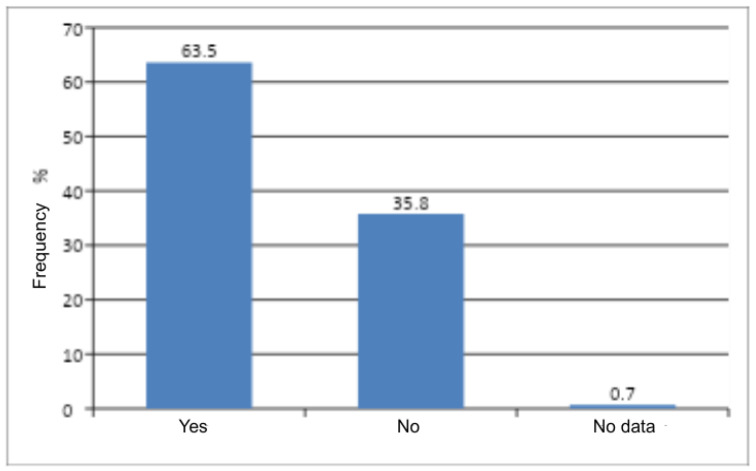
Obstetric history among women.

The Pearson chi-square test revealed a statistically significant difference between the number of women who had ever been pregnant and those who had never been pregnant (*p* < 0.0001) (Table 5). Women with a history of pregnancy predominated in the study group (Figure 3). Obstetric history was found to influence the occurrence of genital discomfort—women with a history of pregnancy experienced genital discomfort more frequently.

#### 3.2.3. Is There a Relationship Between the Occurrence of Genital Discomfort and BMI?

**Figure 4:**
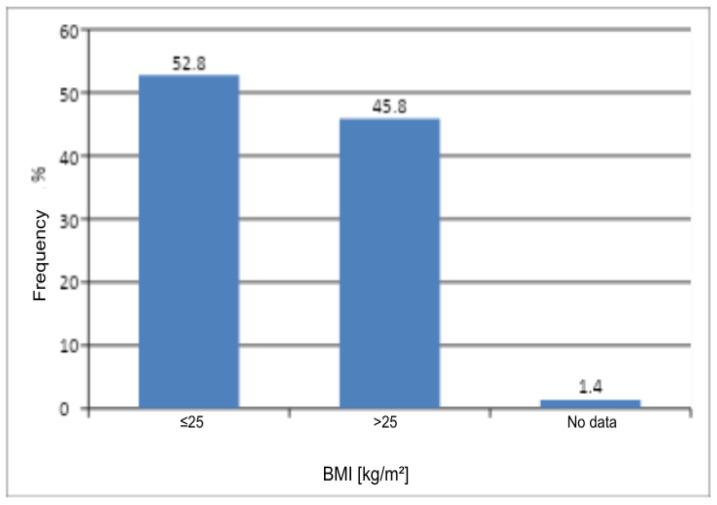
Distribution of BMI in the study group.

The Pearson chi-square test did not reveal a statistically significant difference between the number of women with normal weight or underweight and those with overweight or obesity (*p* > 0.05) (Table 6) (Figure 4). BMI was not found to influence the occurrence of genital discomfort in the studied group of patients.

#### 3.2.4. Is There a Relationship Between the Occurrence of Genital Discomfort and Menopausal Status (Before or After the Last Menstrual Period)?

**Figure 5:**
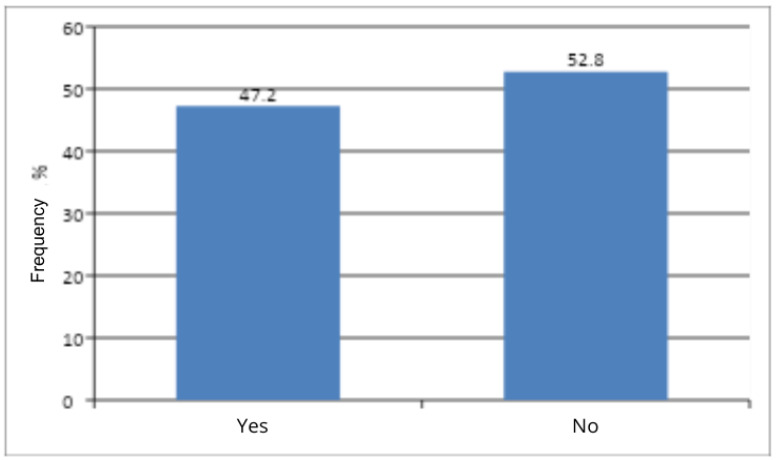
Menopausal status in the study group.

Women were divided into two groups according to menopausal status: Yes (aged 48 years and older) and No (under 48 years of age) (Figure 5). The Pearson chi-square test did not show a statistically significant difference between the number of women in the menopausal group (≥48 years) and those in the premenopausal group (<48 years) (*p* > 0.05) (Table 7). Menopausal status was not found to influence the occurrence of genital discomfort among the studied patients.

## 4. Discussion

The main limitation of this pilot study is the absence of a control group. As a result, the statistical analysis was based on descriptive statistics and the Pearson chi-square goodness-of-fit test. Due to the absence of a control group, it was not possible to perform the chi-square test of independence or to calculate Cramér’s V as a measure of effect size. For the same reason, it was not possible to use odds ratios. The aim of this pilot study was to identify subpopulations in which the research team will conduct an in-depth statistical analysis. We do not exclude the possibility of adding an equal-sized control group to the original study.

The present pilot analysis highlights that the majority of women presenting with genital discomfort to a gynecological outpatient clinic are diagnosed with non-inflammatory vulvar conditions (N90). Among all patients who presented with genital discomfort over the 7-year period, 82.6% received a diagnosis within the N90 category, indicating a predominantly non-inflammatory problem. Thus, the vast majority of women sought care for a chronic condition with a multifactorial etiology, typically requiring more complex and individualized management than acute inflammatory disorders.

The research team of Teigen et al. (2023) conducted a similar study in which 762 women under the care of a gynecological outpatient clinic were surveyed [8]. Patients with VPD accounted for 17% of the study group, and in 28.4% of cases the cause of pain symptoms was an intimate infection. The authors drew attention to the high proportion of patients with fibromyalgia in this group (16.7%). A marked propensity for the development of a genital pain syndrome was observed in patients with fibromyalgia (OR = 1.8, 95% CI 1.1–3.1). A separate subgroup that attracted the attention of the investigators consisted of patients with sexual trauma, specifically a history of childhood sexual abuse [8].

In the Polish patient population, fibromyalgia is a disease entity that often remains underdiagnosed for a long time, and physicians’ knowledge of this condition is not satisfactory [9]. This is problematic due to the significant comorbidity of fibromyalgia with chronic gynecological diseases. In a systematic review by Thornton and Magali (2020), the authors demonstrate that fibromyalgia is associated with a higher prevalence of pelvic floor dysfunction, voiding and defecation disorders, pelvic pain, and gynecological symptoms [10].

In this pilot study, BMI showed no significant association with prevalence of VPDs (*p* = 0.2353). This finding is consistent with the results of other research groups. In the DATRIV study, Harni et al. analyzed a total of 328 women, evenly divided into four groups: those with a normal vulvar appearance (control group), those with impaired vulvar skin, those with vulvodynia, and those with chronic genital discomfort secondary to vulvar dermatoses (including ICD-10 code N90). In the analysis of body weight, it was shown that only patients with vulvar dermatoses had a significantly higher body weight and BMI (mean weight 71.3 kg, BMI 25.4 kg/m^2^) compared with the remaining three groups, in which BMI remained within the normal range (21.6–22.3 kg/m^2^) and did not differ statistically between them [11].

In the previously mentioned cross-sectional study by Teigen et al., women with VPDs had a slightly lower mean BMI than those without pain (25.1 vs. 26.3 kg/m^2^), although both remained within the range typical of the general population. The authors did not interpret BMI as a clinically relevant explanatory factor for chronic vulvar pain; instead, chronic vulvar pain was more strongly associated with indicators of generalized pain sensitization and adverse psychosocial factors [8].

In the present study, it was demonstrated that obstetric history is a statistically significant factor (*p* < 0.0001) and increases the risk of vaginal discomfort. This may result from the fact that, in the postpartum period, infectious conditions of the vagina and/or vulva frequently occur and are easily overlooked due to scant symptoms. As a consequence, these infections may become chronic [12]. In the study by Govender et al. (2024), 184 young postpartum women (between the 6th and 14th week of the puerperium) were examined [13]. Vaginal swabs were collected from all participants to test for Chlamydia trachomatis, Neisseria gonorrhoeae, Trichomonas vaginalis, HSV-2, and to assess bacterial vaginosis (BV) according to Nugent criteria. The results showed that one third of the women were diagnosed with BV [13]. In the context of premalignant conditions of vulvar cancer, and, in particular dysplasia, some authors point out that pregnancy creates an environment that favors the persistence of HPV infection. It is speculated that immunomodulation during pregnancy may disrupt the natural elimination of the virus [14]. HPV is closely associated with the pathogenesis of neoplasia of the vulva and vagina, and since the immunological adaptation of the pregnant woman “probably favors” viral persistence, this may explain the more frequent inflammatory conditions of the vulva in patients with an obstetric history. It should be noted that studies on the impact of pregnancy on the development of HPV-dependent cancers are inconclusive, and there is a need to design a large cohort study.

In this pilot study, the age group most at risk of chronic genital discomfort was 41–50 years. This appears to be consistent with the findings of other research groups [15,16,17,18]. In the cross-sectional study by Geo Celestin Danny et al. (2023), 120 women aged 18 to 78 years presenting to a dermatology outpatient clinic with non-venereal genital diseases were included [18]. The mean age of the participants was 43.08 years (SD 13.79). The authors identified a total of 21 different disease entities; the most common were infectious dermatoses (50% of cases), followed by inflammatory dermatoses (27.5%), conditions classified as “miscellaneous” (15.83%), benign/physiological changes (5.83%), and a single case of malignant neoplasm. The most frequent single diagnosis was vulvovaginal candidiasis (17.5% of the entire sample). These results support the observation that non-venereal diseases of the vulva, including both inflammatory and infectious conditions as well as potentially premalignant dermatoses, are concentrated mainly in middle-aged women [18].

The present study showed no significant association between menopausal status and the occurrence of vaginal discomfort (*p* = 0.3458). This is a surprising finding, given the well-documented association in the literature between menopause and vulvar atrophy [19]. According to the research team of Mitchell et al. (2021), an estimated half of postmenopausal women report symptoms of vulvovaginal discomfort, which significantly impair sexual function and quality of life [20]. On the other hand, referring to the previously cited studies [15,18], the age group most at risk of chronic vulvar disease comprised women in their forties and fifties. Taking into account that the mean age at menopause in Poland is 50–51 years [21], this appears to be consistent with the results of the present study. A limitation of the study that may have influenced the final result was the predominance of premenopausal women who were recruited (47.2% to 52.8%). The research team emphasizes that, for these findings, a more in-depth statistical analysis is warranted.

The last factor that predisposed to the occurrence of genital discomfort was comorbidity with other chronic diseases (metabolic, psychiatric, thyroid, cardiovascular, neurological, pulmonological, gastroenterological, nephrological) (*p* < 0.0001). Due to the pilot nature of the study, the research team adopted a descriptive aim for the statistical analysis, in order to narrow down the directions of analysis in the original study. We plan to extend the statistical analysis in the future to determine significant correlations between diagnoses in the N90 and N76 categories and other chronic diseases; however, this goes beyond the scope of a pilot report.

## 5. Conclusions

1. Genital discomfort is an important issue not only in the field of gynecology, but one that also requires close collaboration with specialists in dermatology, internal medicine, diabetology, dietetics, and, in some cases, oncology.
2. The age group most affected by chronic genital discomfort in this pilot study was 41–50 years, which is consistent with external data.
3. BMI did not show a significant association with the prevalence of VPDs.
4. Obstetric history emerged as a statistically significant factor increasing the risk of vaginal discomfort, which may be related to the high frequency of often oligosymptomatic postpartum vaginal and vulvar infections and to pregnancy-related alterations in immune response and HPV persistence.
5. No significant association was found between menopausal status and vaginal discomfort.
6. Comorbidity with other chronic diseases was strongly associated with the occurrence of genital discomfort, supporting the holistic perspective.

## Figures and Tables

**Table 1:** Statistics of age for female patients.

n	Age (Years)						
	Mean	SD	Median	Q25	Q75	Min.	Max.
288	47.4	20.3	46.0	30.5	67.5	11	91

**Table 2:** Number and frequency of female patients by diagnosis (ICD-10 Code).

Diagnosis		n	%
N76		50	17.4
including	N76.0	10	3.5
	N76.1	8	2.8
	N76.2	1	0.3
	N76.4	7	2.4
	N76.5	3	1.0
	N76.6	5	1.7
	N76.8	16	5.6
N90		238	82.6
including	N90.0	5	1.7
	N90.1	2	0.7
	N90.2	15	5.2
	N90.3	10	3.5
	N90.4	8	2.8
	N90.5	7	2.4
	N90.6	18	6.3
	N90.7	5	1.7
	N90.8	27	9.4
	N90.9	141	49.0
All		288	100

**Table 3:** Number and frequency of women by age and the result of Pearson’s Chi-Square Test of Independence.

Age	n	%	χ^2^	df	*p*
11–20	31	10.8	30.56	7	<0.0001
21–30	41	14.2			
31–40	45	15.6			
41–50	47	16.3			
51–60	36	12.5			
61–70	36	12.5			
71–80	44	15.3			
81–91	8	2.8			
All	288	100			

χ^2^—chi-square statistic; df—degrees of freedom; p—probability level (*p*-value).

**Table 4:** Number and frequency of women by the presence of comorbidities and the result of Pearson’s Chi-Square Test of Independence.

Comorbidities	n	%	χ^2^	df	*p*
Yes	203	70.5	48.35	1	<0.0001
No	85	29.5			
All	288	100			

χ^2^—chi-square statistic; df—degrees of freedom; p—probability level (*p*-value).

**Table 5:** Number and frequency of women by the presence of obstetric history and the result of Pearson’s Chi-Square Test of Independence.

History of Pregnancy	n	%	χ^2^	df	*p*
Yes	183	63.5	22.38	1	<0.0001
No	103	35.8			
No data	2	0.7			
All	288	100			

χ^2^—chi-square statistic; df—degrees of freedom; p—probability level (*p*-value).

**Table 6:** Number and frequency of women by BMI and the result of Pearson’s Chi-Square Test of Independence.

BMI	n	%	χ^2^	df	*p*
<25	152	52.8	1.41	1	0.2353
>25	132	45.8			
No data	4	1.4			
All	288	100			

χ^2^—chi-square statistic; df—degrees of freedom; *p*—probability level (*p*-value).

**Table 7:** Number and frequency of women by menopausal status and the result of Pearson’s Chi-Square Test of Independence.

Menopausal Status	n	%	χ^2^	df	*p*
Yes	136	47.2	0.89	1	0.3458
No	152	52.8			
All	288	100			

χ^2^—chi-square statistic; df—degrees of freedom; *p*—probability level (*p*-value).

## Data Availability

The original data presented in the study are openly available in Zenodo at: https://doi.org/10.5281/zenodo.17469511 (published on 28 October 2025).

## Abbreviations

The following abbreviations are used in this manuscript:

VPD	vulvar pain disorders
BMI	body-mass index
BV	bacterial vaginosis
